# Supplementary material for: Interferon mediated prophylactic protection against respiratory viruses conferred by a prototype live attenuated influenza virus vaccine lacking non-structural protein 1
Source: Sci Rep. 2021 Nov 12;11:22164. doi: 10.1038/s41598-021-01780-8 (PMC8589955; doi:10.1038/s41598-021-01780-8)
Supplement: Supplementary file 1 — Supplementary Information. [file 41598_2021_1780_MOESM1_ESM.docx]

**Supplementary Table 1.** Units of IFN present in the allantoic fluid of 10-days embryonated chicken eggs which were inoculated with WT A/PR8/34 or ΔNS1 influenza A viruses.

| **Virus^a^** | **Egg number** | **IFN (Uml^-1^)^b^** |
| --- | --- | --- |
| **rWT PR8** | 1 | <16 |
|  | 2 | <16 |
| **ΔNS1** | 3 | 400 |
|  | 4 | 400 |
| **Mock** | 5 | <16 |
|  | 6 | <16 |

^a^Eggs were inoculated with 10^3^ PFU of rWT-PR8 or ΔNS1 virus

^b^Amount of IFN in the allantoic fluid was measured 18 hours post inoculation

**Supplementary Table 2.** Viral titers, bodyweight changes and lung weights in A2G mice infected with ΔNS1 and hvPR8 viruses.

| **Virus** | **Day 3** | | | | **Day 6** | | | |
| --- | --- | --- | --- | --- | --- | --- | --- | --- |
|  | **Mouse** | **PFU/ml^a^** | **Bodyweight changes ^b^** | **Lung weight** | **Mouse** | **PFU/ml^a^** | **Bodyweight changes ^b^** | **Lung weight** |
| **ΔNS1^c^** | 1 | <10 | +0.73 g | 0.13 g | 7 | <10 | +1.59 g | 0.14 g |
|  | 2 | <10 | +1.19 g | 0.13 g | 8 | <10 | +2.89 g | 0.15 g |
| **hvPR8^e^** | 3 | 3 x 10^8^ | -3.05 g | 0.32 g | 9 | 3 x 10^6^ | -6.65 g | 0.34 g |
|  | 4 | 3 x 10^7^ | -3.09 g | 0.27 g | 10 | 7 x 10^6^ | -4.69 g | 0.24 g |
| **ΔNS1+ hvPR8^d^** | 5 | 2 x 10^4^ | +1.58 g | 0.15 g | 11 | <10 | -0.03 g | 0.12 g |
|  | 6 | 6 x 10^2^ | -0.42 g | 0.11 g | 12 | 1 x 10^4^ | +0.37 g | 0.11 g |

^a^ Lungs were homogenized in 2 ml of PBS and viral titers were determined by standard plaque assay

^b^ Numbers represent differences with respect to the bodyweight at the time of infection

^c^ Animals were treated intranasally on day 0 with 2x10^5^ PFU of ΔNS1 virus

^d^ Animals were treated intranasally on day -1 with 2x10^5^ PFU of ΔNS1 virus followed by intranasal challenge on day 0 with 2x10^4^ PFU of hvPR8 virus

^e^ Animals were infected intranasally on day 0 with 2x10^4^ PFU of hvPR8


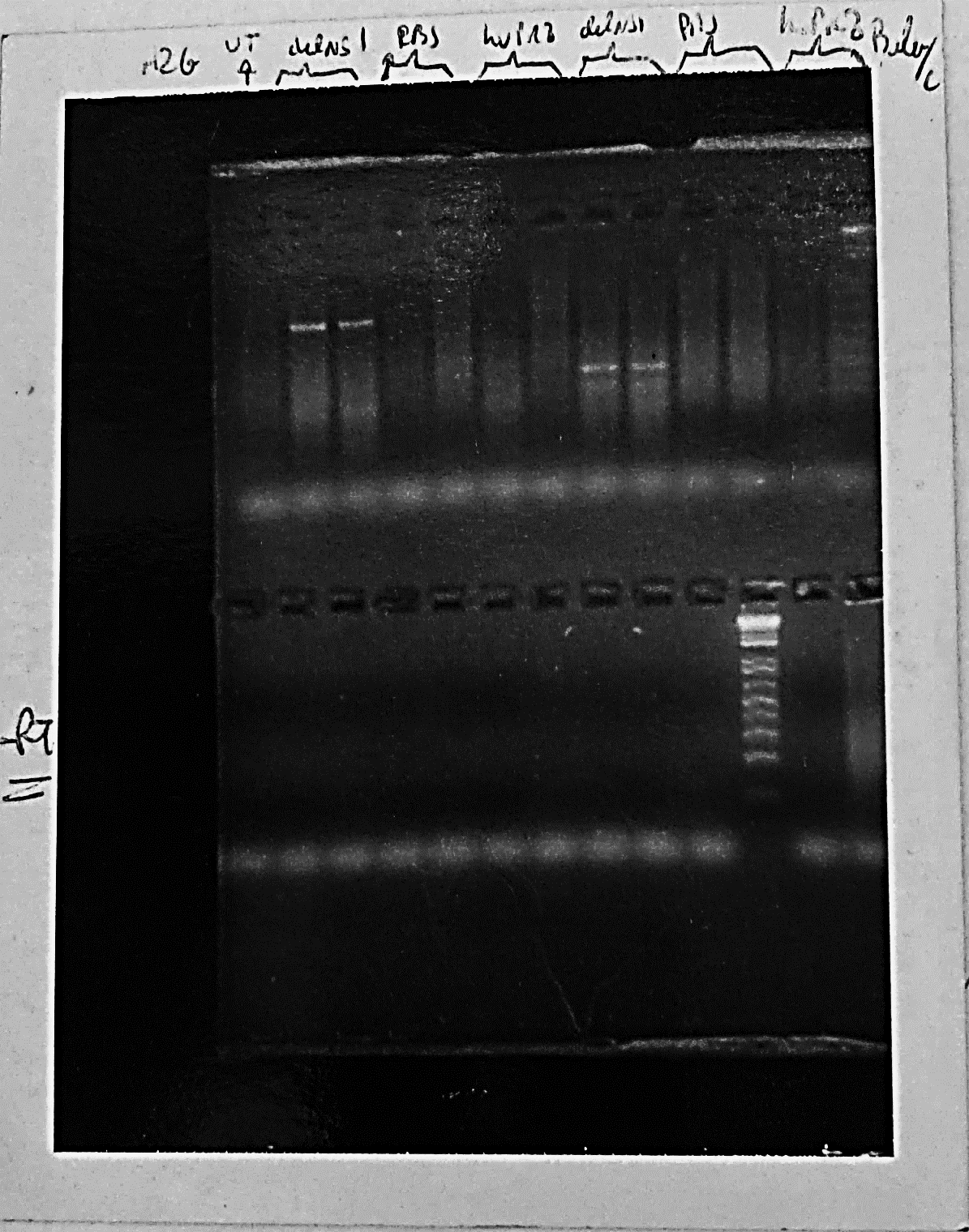


**A2G-Mx1**

**BALB/c-WT**

**UT**

**ΔNS1**

**PBS**

**hvPR8**

**ΔNS1**

**PBS**

**hvPR8**

**Supplementary 1. Dose dependent pre-treatment of ΔNS1 protects A2G-Mx1 mice but not wild-type C57BL/6 from a lethal hvPR8 virus challenge.** Full size gel image corresponding to figure 3A. Groups of two A2G or BALB/c mice were intranasally treated with PBS or 2.5x10^5^ PFU of ΔNS1 hvPR8 influenza viruses. 24 hours post challenge, total RNA present in lung tissues were extracted and were used for RT-PCR reactions using *Mx1* specific primers. PCR products were run in an agarose gel.
